# Integrated Metabolomics and Network Pharmacology to Establish the Action Mechanism of Qingrekasen Granule for Treating Nephrotic Syndrome

**DOI:** 10.3389/fphar.2021.765563

**Published:** 2021-12-06

**Authors:** Yanfen Duan, Dongning Zhang, Yan Ye, Sili Zheng, Ping Huang, Fengyun Zhang, Guoyan Mo, Fang Huang, Qiang Yin, Jingjing Li, Lintao Han

**Affiliations:** ^1^ Faculty of Pharmacy, Hubei University of Chinese Medicine, Wuhan, China; ^2^ College of Basic Medical Sciences, Hubei University of Chinese Medicine, Wuhan, China; ^3^ Key Laboratory of Traditional Chinese Medicine Resource and Prescription, Ministry of Education, Wuhan, China; ^4^ Xinjiang Uygur Pharmaceutical Co., Ltd., Urumqi, China

**Keywords:** metabolomics, molecular docking, nephrotic syndrome, network pharmacology, qingrekasen granule

## Abstract

Nephrotic syndrome (NS) is a clinical syndrome resulting from abnormal glomerular permeability, mainly manifesting as edema and proteinuria. Qingrekasen granule (QRKSG), a Chinese Uyghur folk medicine, is a single-flavor preparation made from chicory (Cichorium intybus L.), widely used in treating dysuria and edema. Chicory, the main component in QRKSG, effectively treats edema and protects kidneys. However, the active components in QRKSG and its underlying mechanism for treating NS remain unclear. This study explored the specific mechanism and composition of QRKSG on an NS rat model using integrated metabolomics and network pharmacology. First, metabolomics explored the relevant metabolic pathways impacted by QRKSG in the treatment of NS. Secondly, network pharmacology further explored the possible metabolite targets. Afterward, a comprehensive network was constructed using the results from the network pharmacology and metabolomics analysis. Finally, the interactions between the active components and targets were predicted by molecular docking, and the differential expression levels of the target protein were verified by Western blotting. The metabolomics results showed “D-Glutamine and D-glutamate metabolism” and “Alanine, aspartate, and glutamate metabolism” as the main targeted metabolic pathways for treating NS in rats. AKT1, BCL2L1, CASP3, and MTOR were the core QRKSG targets in the treatment of NS. Molecular docking revealed that these core targets have a strong affinity for flavonoids, terpenoids, and phenolic acids. Moreover, the expression levels of p-PI3K, p-AKT1, p-mTOR, and CASP3 in the QRKSG group significantly decreased, while BCL2L1 increased compared to the model group. These findings established the underlying mechanism of QRKSG, such as promoting autophagy and anti-apoptosis through the expression of AKT1, CASP3, BCL2L1, and mTOR to protect podocytes and maintain renal tubular function.

## 1 Introduction

Nephrotic syndrome (NS) is a clinical condition caused by inflammation, oxidative stress, immune injury, and podocyte damage in the kidney (Imig and Ryan, 2013; Zhang et al., 2018). The disease is characterized by edema, proteinuria, hypoalbuminemia, and hyperlipidemia, due to abnormal glomerular permeability (Wang and Greenbaum, 2019; Tsuji et al., 2020). The current treatments for NS are extensive, repetitive, non-specific, and have side effects because of the understanding of the biological processes leading to NS(Saleem, 2019). Thus, NS is often accompanied by various complications, complicated treatment procedures, and unsatisfactory curative effects. Eventually, NS develops into renal failure, which negatively impacts livelihoods and the economy (Wang et al., 2000). Considering the far-reaching impacts of NS, it is essential to establish NS-related mechanisms and suitable drugs. Recently, many patients with kidney diseases have resorted to traditional Chinese medicines (TCM) (Zhao et al., 2015), whose biological activity and therapeutic effects are proven *in vitro* and animal experiments (Fang et al., 2013; Zhang et al., 2014). Chicory (Cichorium intybus L.), is a medicinal plant widely distributed in Europe, America, and Asia, and the main components of Qingrekasen Granule (QRKSG), listed in the Pharmacopoeia of the People’s Republic of China (2020 edition). The plant functions as a diuretic, anti-inflammatory, digestive, cardiotonic, and liver tonic (Petrovic et al., 2004; Satmbekova et al., 2018). Moreover, the sesquiterpene lactones 8-Deoxylactucin, lactucin, and lactucopicrin in chicory extract inhibit the production of prostaglandin E2 (PGE2) and COX-2 protein expression (Cavin et al., 2005), hence, reducing incidences of edema and inflammatory pain (Wesolowska et al., 2006). For example, the chicory extract reduces carrageenan-induced paw edema (Rizvi et al., 2014). The extract significantly reduces α1-microglobulin in urine, improves histological appearance, and repairs kidney damage (Pourfarjam et al., 2017). QRKSG is a granule made from chicory, and is approved by the China Food and Drug Administration, approval number (CFDA approval no. Z65020172). However, the functional mechanisms and value of QRKSG in NS are unknown.

Metabolomics and network pharmacology are two disciplines for understanding the mechanisms of drug action by linking the metabolites to disease targets. Metabolomics detect metabolic changes (Majumder et al., 2021) and the positive role of metabolites in physiology and disease development in organisms (Rinschen et al., 2019). In TCM, metabolomics revealed the mechanisms of various prescriptions, including the Qi-Dan Fang Granule (Wu et al., 2014) and the Danggui-Shaoyao-San (Wang et al., 2020). Meanwhile, network pharmacology predicts the possibility of drug compound-disease target combinations and pathway analysis (Xiong et al., 2019; Xiong et al., 2020). Thus, the approach is suitable for analyzing TCM preparations (Zhang et al., 2019). For example, a new method of treating osteoarthritis has been established using network pharmacological analysis (Zhu et al., 2019) thus, strengthening evidence-based research and promoting the development of high-quality Chinese medicine (Li and Zhang, 2013).

**FIGURE 1:**
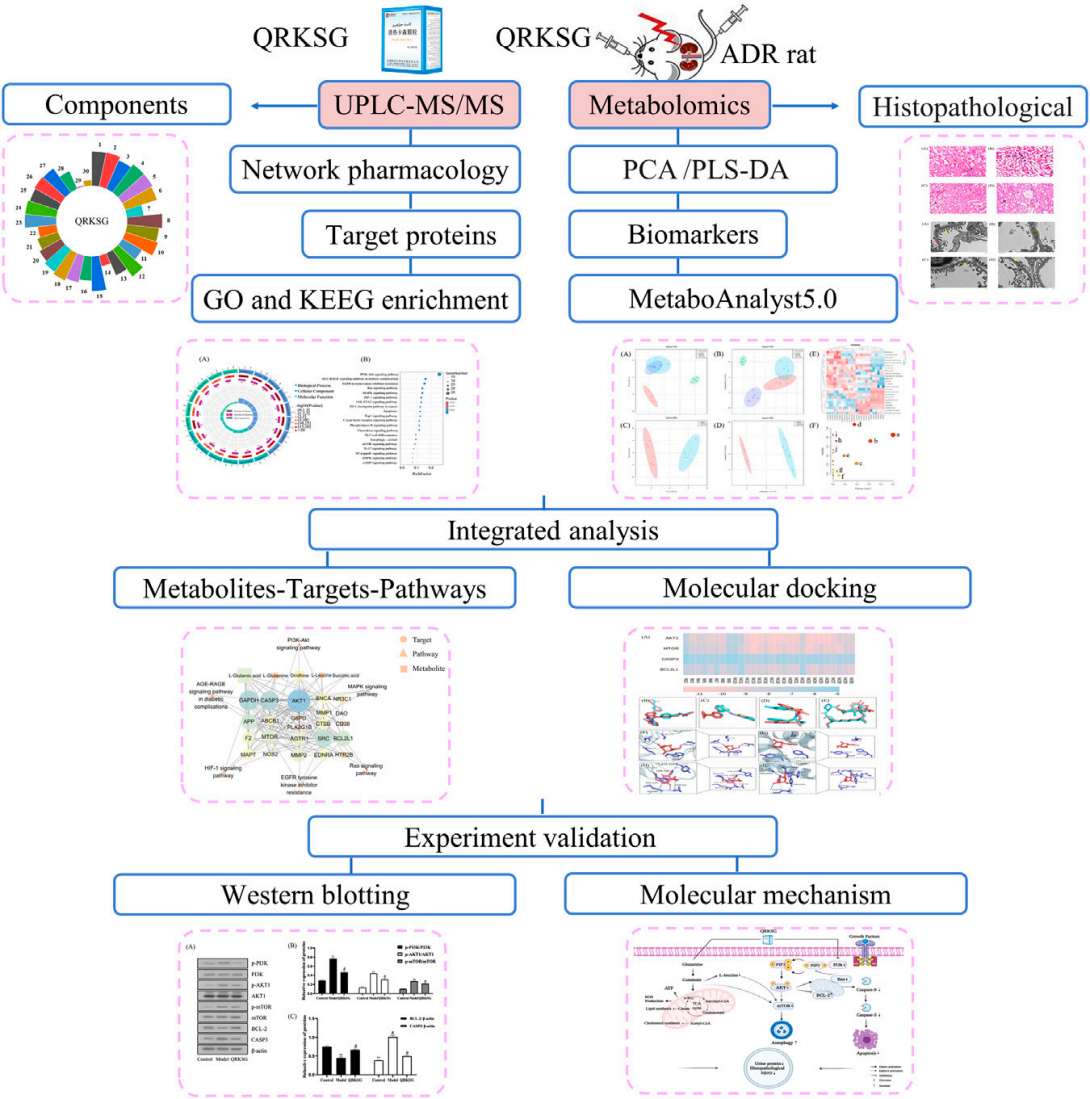
Flow chart of the research process.

The findings in this research form a basis for clinical approval and QRKSG use for managing NS. The flow chart of the research process is shown in Figure 1.

## 2 Materials and Methods

### 2.1 Reagents and Materials

The Qingrekasen Granule was purchased from Xinjiang Uygur Pharmaceutical Co., Ltd. (NO.1807521, Xinjiang, China). Adriamycin (ADR) was purchased from Shenzhen Main Luck Pharmaceuticals Inc. (No. 2007E1, Shenzhen, China), and Benazepril was purchased from Novartis (No. 2007, Beijing, China). The BCA Protein Assay Kit was obtained from Beyotime Biotech Inc. (P0012, Shanghai, China), and Guangzhou Jet Bio-Filtration Co., Ltd. (Guangzhou, China) provided the Elisa plates (12 well strip ×8). HPLC grade methanol and acetonitrile were purchased from Merck KGaA (Darmstadt, Germany). Anti-PI3K and anti-p-PI3K antibodies were purchased from Cell Signaling Technology Inc. (MA, United States). The Anti-AKT, anti-p-AKT, anti-mTOR, anti-P-mTOR, anti-BCL-2, anti-Caspase-3, and Beta-actin antibodies were purchased from Bioworld Technology Inc. (MN, United States). All other reagents were HPLC grade.

### 2.1 *In vivo* Experiment

Thirty-two male SD (Sprague Dawley) rats (180–220 g) were obtained from Sibefu (Beijing) Biotechnology Co., Ltd. (YFY-2018–0,002, Beijing, China). The SD rats were housed in an environmentally conditioned room with a 12 h light/dark cycle, at 25 ± 2°C, and 50 ± 5% relative humidity. All rats accessed drinking water and feed *ad libitum*. The protocols in this study strictly followed the animal ethics standards of the Hubei University of Chinese Medicine. After 1 week of acclimation, the rats were randomly assigned into four groups, including the control (*n* = 8) and ADR (7 mg/kg) treatments [three groups (*n* = 24)].

The NS model was induced by injecting normal saline through the tail vein (Frenk et al., 1955; Korzets et al., 1997). A 24 h urine protein test was performed once a week for four experimental weeks. Mineral oil was added to the urine collection container to avoid evaporation. After successfully inducing the NS model, the ADR-treated rats were randomly assigned into three groups (*n* = 8 per group): model, QRKSG (treated with a clinically equivalent dose of 1.62 g/kg/d), and benazepril (treated with a clinically equivalent dose of 0.9 mg/kg/d) groups. The control and model groups were treated with normal saline. All treatments were gavage administered once per day for 4 weeks, after which the animals were anesthetized, their kidneys were removed, and blood was collected from the abdominal aorta using a syringe.

### 2.3 Histopathological Analysis

One portion of the left kidney tissue from each rat was fixed in 4% paraformaldehyde impregnated wax, paraffin-embedded, and serially sectioned (4–7 μm). After staining, the kidney sections were imaged and photographed with a biomicroscope (Olympus BX53, Olympus Optical Co., Tokyo, Japan). Another portion of left kidney tissue (<1 mm^3^) was fixed and post-fixed using 2.5% glutaraldehyde and 1% osmium tetroxide, respectively. The tissues were sequentially treated by ethanol acetone dehydration and acetone embedding infiltration overnight, embedded, sectioned (60–80 nm), uranium-lead double-stained, and finally observed by transmission electron microscopy (HT7700, Hitachi High-Technologies Corporation, Tokyo, Japan). Right kidney tissues were washed with saline, freeze-dried in liquid nitrogen on filter paper for 30 min, and stored in the refrigerator at −80°C.

### 2.4 Urine and Blood Chemistry

The collected blood samples were incubated at 4°C for 30 min, and the serum was separated by centrifugation (3,000 rpm for 10 min) for subsequent analysis. A biochemical analyzer (Chemray240, Rayto, Guangdong, China) detected albumin (ALB), total triglycerides (TG), creatinine (Cr), total cholesterol (TC), total protein (TP), and urea nitrogen (BUN) levels in the serum. In addition, the urine samples were centrifuged, and the collected supernatant was stored at −80°C for further analysis. The 24 h urine total protein concentration was determined using the BCA Protein Assay Kit and microplate reader (Epoch, Bio Tek, VT, United States) following the manufacturer’s instructions.

### 2.5 Gas Chromatography-Mass Spectrometry (GC-MS) Analysis of Metabolites in Kidney Samples

#### 2.5.1 Kidney Sample Preparation

The right kidney tissues were thawed at room temperature. Approximately 600 μL of the right kidney tissue homogenate was extracted with methanol, vortexed for 1 min, and ultrasonically centrifuged for 10 min in centrifuge tubes sitting in an ice bath to obtain the metabolites. Afterward, the supernatant was concentrated through centrifugation and evaporated to dryness. The internal standards: 10 μL D27-myristic acid (0.75 mg/ml) and 40 μL methoxyamine pyridine solution (40 mg/ml) were then added and maintained for 90 min. Finally, 80 μL of MSTFA+1% TMCS (Sigma-Aldrich, MO, United States) and 20 μL n-hexane were added for derivatization and derivatization reaction termination. After centrifugation, 1 μL of the supernatant was transferred to a micro bottle for subsequent analysis.

#### 2.5.2 GC-MS Data Acquisition

The GC-MS was performed as previously described (Cao et al., 2020), using Gas Chromatography-Mass Spectrometer (7,890B–5977B, Agilent Technologies, CA, United States) equipped with a DB-5MS quartz capillary column (0.25 mm × 30 mm, 0.25 μm). Analysis was performed using 1 µL injection volume with helium as the carrier gas (99.999%). The flow rate was 1.0 ml/min and a split ratio of 10:1. The column temperature was maintained at 60°C for 1 min, then increased at a rate of 10°C/min up to 250°C and maintained at 250°C for 10 min. The injection temperature was 250°C, while that of the electron bombardment ion source was 230°C, with a solvent delay of 5.9 min and a scanning range of the mass spectrum at m/z 50.0–600.0.

### 2.6 Metabolites and Metabolic Pathway Analysis

The differential metabolites were identified by converting the GC-MS experimental data into compound retention time and relative peak area information using the Agilent Masshunter software. The online tool, MetaboAnalyst5.0 (Chong et al., 2018; Chong et al., 2019) (version 5.0), performed the principal component analysis (PCA) and partial least squares discriminant analysis (PLS-DA). The Pathway Analysis project in the online tool MetaboAnalyst5.0 analyzed metabolic pathways. Specified path analysis parameters included the visualization method using a scatter plot (testing significant features), the enrichment method using the hypergeometric test, the topology analysis approach using relative-betweenness centrality, and the reference metabolome technique using all compounds in the pathway library. Metabolites with *p* < 0.05 and VIP>1.0 were considered as potential biomarkers. The network analysis project in the MetaboAnalyst5.0 online tool performed the comprehensive metabolomic and network pharmacology analysis. Differential metabolites and differential metabolic genes were sequentially imported for analysis. Selected gene-metabolite interaction networks in the networks analysis options obtained the interaction between metabolites and targets.

### 2.7 UPLC-MS/MS for QRKSG Component Analysis

#### 2.7.1 UPLC-MS/MS Sample Preparation

A vacuum freeze dryer (Scientz-100F, Zhejiang, China) and grinder (MM 400, Retsch, Shanghai, China) were used to freeze-dry and crush the QRKSG. Afterward, 100 mg of the lyophilized powder was dissolved in methanol, vortexed six times, and refrigerated overnight at 4°C. The extracts were filtered for analysis on the UPLC-MS/MS system.

#### 2.7.2 UPLC-MS/MS Component Identification

An integrated UPLC (NexeraX2, Shimadzu, Japan) and MS/MS (4500QTRAP, Applied Biosystems, MA, United States) performed the QRKSG qualitative analysis. The chromatographic separation was performed at room temperature on a chromatographic column (181.8 µm, 2.1 mm*100 mm, Agilent). For chromatographic analysis, the mobile phase A and B were pure water/0.1% formic acid and acetonitrile with a 0.35 ml/min flow rate. The flow was programmed to start with 95% A and 5% B; 1–9 min, 5%–95% B; 9–10 min 5% A, 95% B; 9–10 min 95% B; 10–11 min, 5.0% B; 11–14 min, 5.0% B. The column oven was set at 40°C, and the injection volume was 4 µL. The mass spectrometry conditions were: ion source, vortex spray, and element temperature at 550°C; spray voltage - positive ion mode (5,500 v)/negative ion mode (−4500 V); 50psi (GSI), 60psi (GSII), 25psi (CUR).

### 2.8 Network Pharmacology Analysis

First, the QRKSG biological targets were obtained from the SwissTargetPrediction database (http://www.swisstargetprediction.ch/). Secondly, the NS biological targets were obtained from GeneCards (https://www.genecards.org/), a searchable and integrated human gene database (Safran et al., 2010). Finally, the QRKSG target information for treating NS was obtained by interacting with the above two. The DAVID database (https://david.ncifcrf.gov/summary.jsp) was used as the functional annotation of gene ontologies (GO) and Kyoto Encyclopedia of Genes and Genomes (KEGG) pathways.

### 2.9 Comprehensive Analysis

The complex relationship of the metabolites-targets-pathways network was constructed using Cytoscape3.8.0 to determine the underlying mechanism of QRKSG on NS.

### 2.10 Molecular Docking

The AKT1, mTOR, CASP3, and BCL2L1 three-dimensional (3D) structures, in. pdb format were obtained from the RCSB PDB database (https://www. rcsb.org/). The AutodockTools1.5.6 software deleted water, cleaned the protein, changed the forcefield on the .pdb file, saved it in .pdbqt format, and was the receptor ready to complete. The ChemOffice and Openbabe 2.3.1L preserved the 2D structure of the QRKSG active ingredient ligands and generated energy-minimized 3D conformations, saved in. pdb format. AutodockTools1.5.6 converted the .pdb files to .pdbqt format, and the ligands were ready for finishing. Autodock Vina1.1.2 was used for molecular docking of the prepared ligands to the receptor. The docking parameters were: AKT1 (4EJN), grid center 35.389 43.721 18.443, size 24.046 24.046 24.046; mTOR (4DRH), grid center −4.934 20.004–1.832, size 24.215 24.215 24.215; CASP3 (2XYG), grid center 36.259 38.656 32.142, size 14.456 14.456 14.456; and BCL2L1 (3ZLN), grid center −18.564–12.299 12.808, size 21.42 21.42 21.42.

### 2.11 Western Blot

The kidney tissues were thawed at 4°C in a refrigerator for protein extraction. Afterward, 20 mg of kidney tissues were homogenized with 200 μL lysis buffer. The total protein concentration was determined using the BCA kit (PICPI23225, Thermo Fisher Scientific, MA, United States) following the manufacturer’s instructions. The proteins were separated using 10% SDS ammonium persulfate gel electrophoresis and transferred into PVDF membranes. The membranes were blocked with 5% skimmed milk in TBST (Tris-buffered saline, 0.1% Tween 20) solution for 1 h at room temperature, followed by overnight incubation with the primary antibody PI3K (1:1,000 dilution), p-PI3K, AKT, p-AKT, mTOR, p-mTOR, BCL-2, Caspase-3 and reference Beta-actin (all 1:1,000 dilution) at 4°C. Next, the membranes were washed in TBST solution and incubated with Anti-Rabbit lgG conjugated to horseradish peroxidase (HRP) at 37°C for 1 h and washed in TSBT solution. The immune response area in the membranes was observed using the enhanced chemiluminescence (ECL) kit (WBKLS0100, Millipore, MA, United States) following the manufacturer’s instruction. The density and immune response analysis was performed using the Tanon-5200 imaging system (Tanon Science and Technology Co. Ltd., Shanghai, China) and ImageJ Software (NIH, MD, United States).

### 2.12 Statistical Analysis

All quantitative group data were analyzed using a one-way analysis of variance (ANOVA) and presented as mean ± standard deviation. *p* < 0.05 was considered statistically significant.

## 3 Results

### 3.1 Serum Biochemical Indexes and Urine Protein Quantitation

**FIGURE 2:**
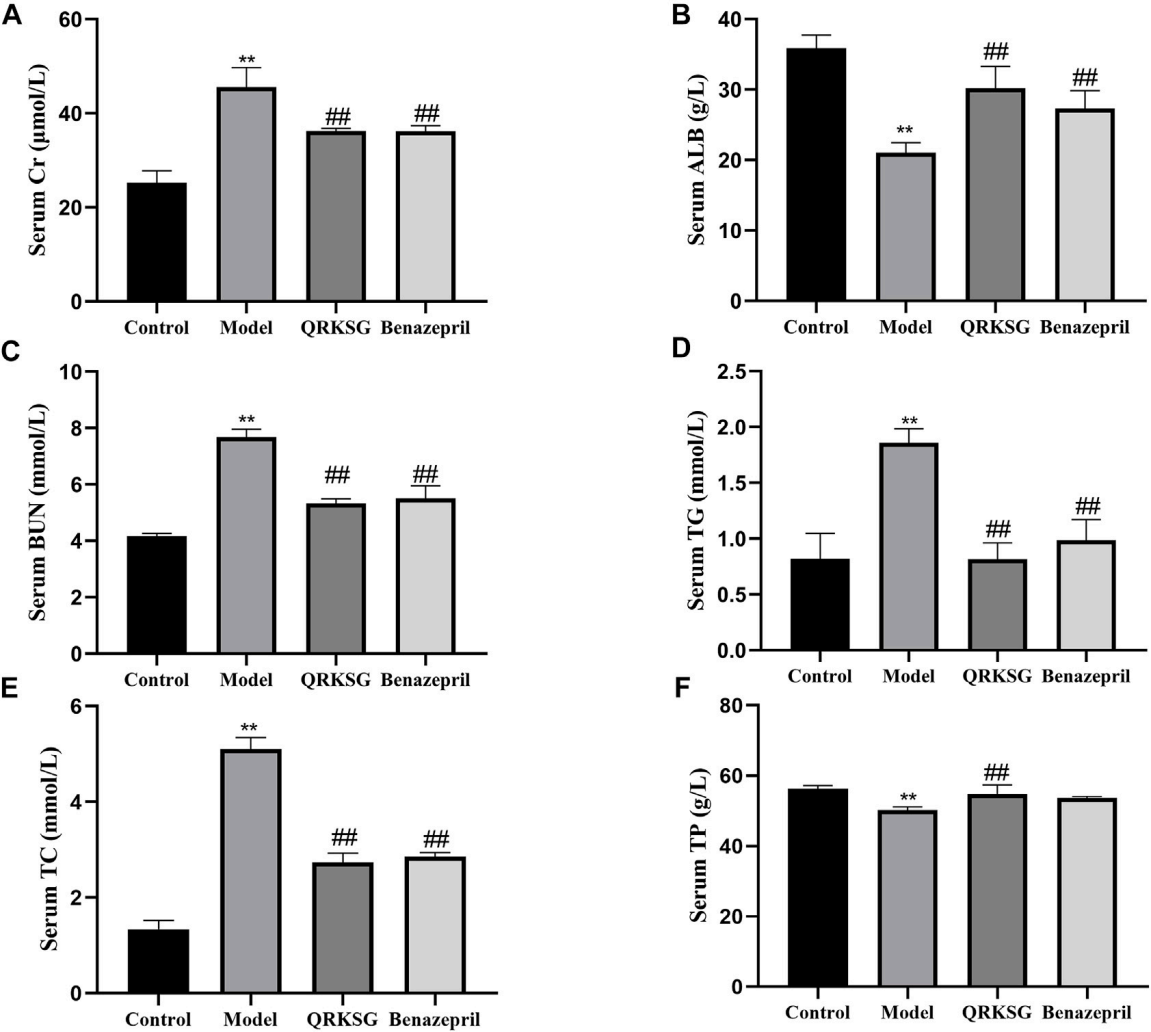
Changes in serum biochemical parameters. **(A)** Serum Cr level **(B)** Serum ALB level **(C)** Serum BUN level **(D)** Serum TG level **(E)** Serum TC level **(F)** Serum TP level. The data are presented as mean ± SD, **p < 0.05 shows comparison against the control group, and ^##^ *p* < 0.05, against the model group.

**FIGURE 3:**
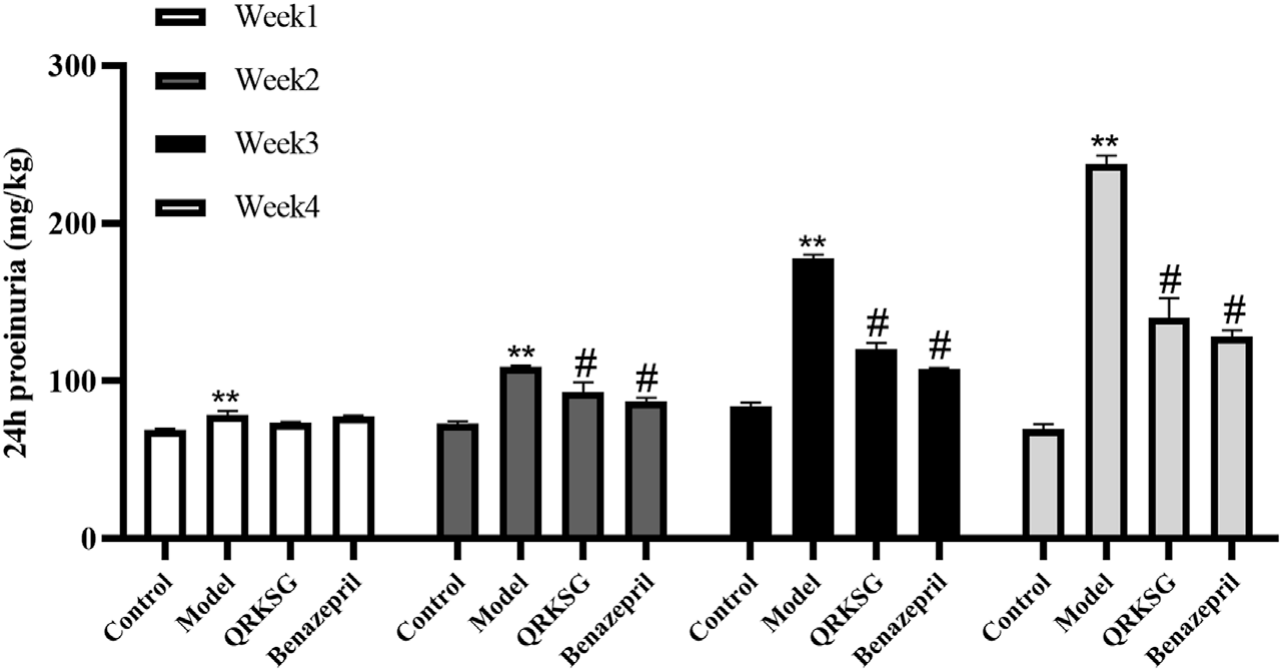
24 h proteinuria in normal and NS model rats. The data are presented as mean ± SD, **p < 0.001 shows comparison against the control group; ^#^ *p* < 0.05 shows comparison against the model group.

The TG, BUN, TC, and Cr levels in the model group serum increased, while ALB and TP decreased more than the control group. However, QRKSG treatment reduced the levels of TG, BUN, TC, and Cr in the serum, while the level of ALB and TP increased (Figure 2). The 24 h proteinuria assessment in the rats revealed that the proteins in urine gradually increased from week 1 to week 4 after the model was established (Figure 3).

In week one, the urine protein content in the control group was significantly lower than the ADR-treated group, indicating that the NS was successfully induced. However, the proteinuria content was not significantly different between QRKSG and benazepril groups in the first week compared to the model group. Nevertheless, the proteinuria content was significantly higher in the model than in the QRKSG group in the 3rd and 4th weeks (*p* < 0.001). In addition, the proteinuria level was higher in the QRKSG than in the benazepril group in the 4th week. However, the urine protein levels were reduced in the QRKSG and benazepril groups compared to the control.

### 3.2 Histopathological Analysis

**FIGURE 4:**
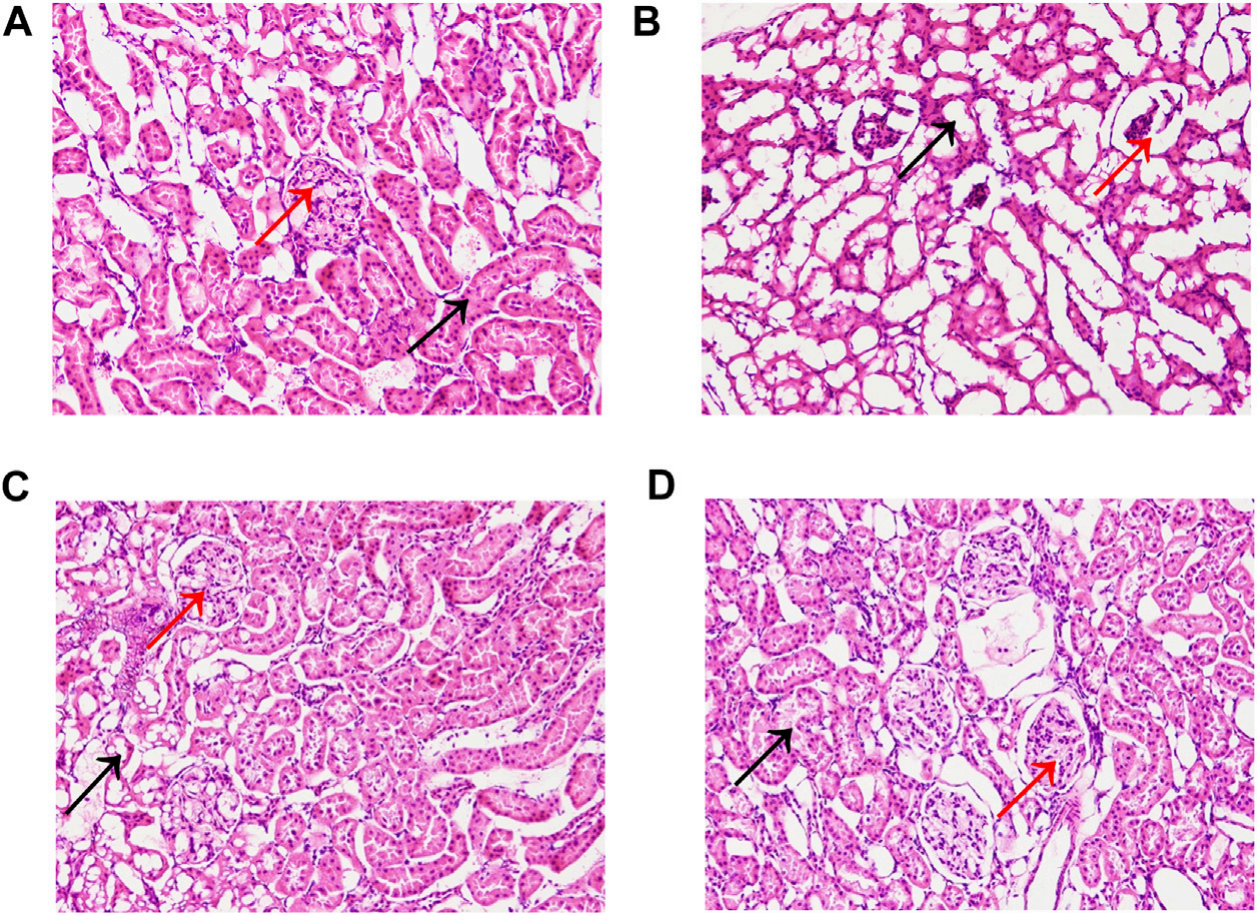
Representative micrographs of histopathology in each group at magnification ×200. Black arrows represent kidney tubules, and red arrows represent glomeruli. **(A)** control group **(B)** model group **(C)** benazepril group **(D)** QRKSG group.

**FIGURE 5:**
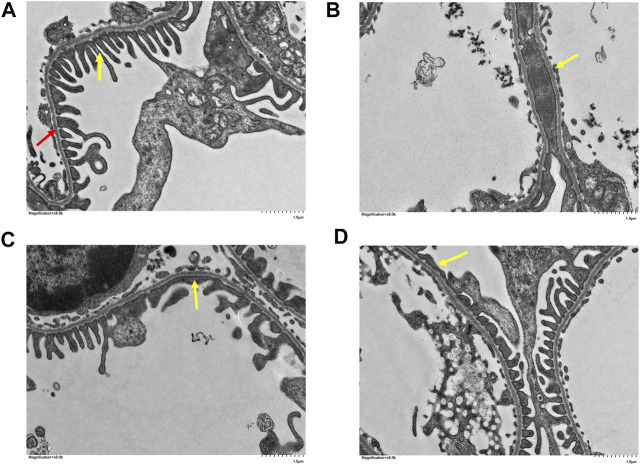
Glomerular foot cell changes under electron microscopy in each group at magnification ×8,000. The yellow arrow points to the foot processes and the red arrow to the basement membrane. **(A)** control group **(B)** model group **(C)** benazepril group **(D)** QRKSG group.

The pathological changes in the kidney tissues were observed under the light microscope (Figure 4). The glomeruli and tubules structures were normal, and the control group had no visible damage. Nevertheless, the model group showed renal tubular abnormalities, dilation of the tubular lumen, and edema. However, the benazepril and QRKSG groups showed significantly reduced edema and morphological changes than the model group. Groups of rat glomerular podocytes were observed by transmission electron microscopy (Figure 5). The results showed that foot processes in the normal group were neatly aligned, structurally intact, and the basement membranes were uniformly thick. The model group had numerous diffused fusions in the foot processes, and the structure of the podocytes changed, consistent with the structural changes of podocytes during NS development (Smoyer and Mundel, 1998). Moreover, the degree of fusion significantly reduced in the QRKSG and benazepril groups, and some normal podocytes were visible. The histological evaluation confirmed that QRKSG alleviates the pathological damage caused by ADR-induced NS in the kidneys of rats.

### 3.3 Effects of QRKSG on Kidney Metabolism

**FIGURE 6:**
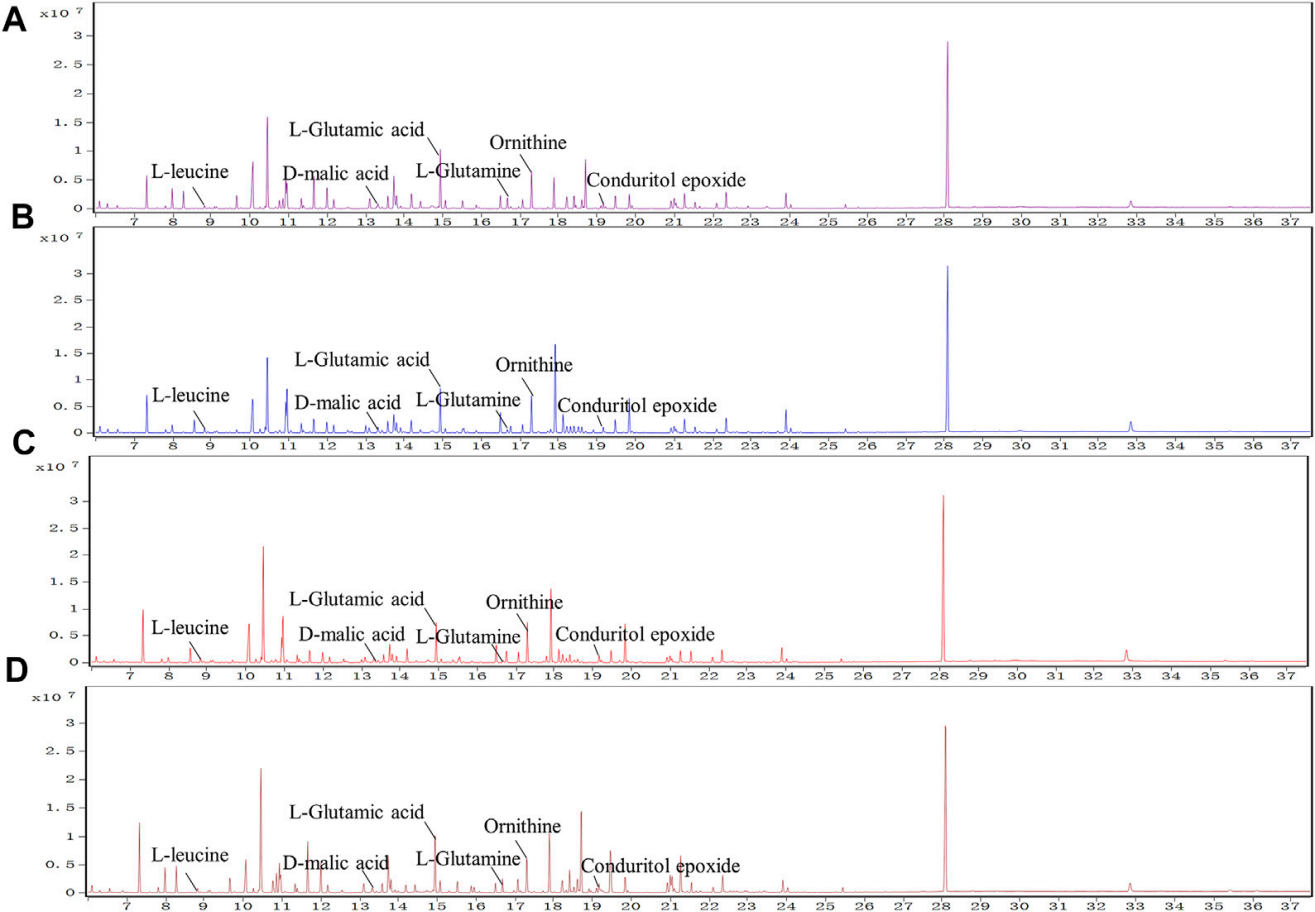
TICs of metabolites from kidneys. **(A)** control group **(B)** benazepril group **(C)** QRKSG group **(D)** model group.

**FIGURE 7:**
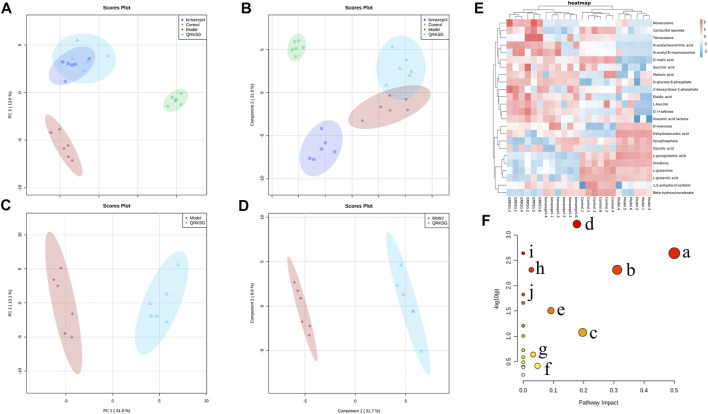
QRKSG intervention on NS rats. **(A)** PCA score chart; **(B)** PLS-DA score chart; **(C)** PCA analysis of the model and QRKSG groups; **(D)** PLS-DA analysis of the model and QRKSG groups; **(E)** Heatmap visualization of the 24 potential metabolites expressed in rat kidneys; **(F)** MetaboAnalyst5.0 pathway analysis.

The kidney samples obtained from the control, model, benazepril, and QRKSG groups were analyzed by GC-MS, and the total ion current chromatograms (TIC) are shown in Figure 6. The model and QRKSG groups showed a considerable degree of separation (Figure 7C). The PLS-DA R^2^ (=0.963) and Q^2^ (=0.885) indicate an excellent PLS-DA model for discrimination and prediction (Figure 7D). The kidney metabolism spectrum analysis by PCA and PLS-DA revealed that the metabolisms in QRKSG and benazepril groups ranged between the model and control groups. These results indicate that treatment caused normal metabolite levels in rat kidneys (Figures 7A,B).

### 3.4 Metabolic Pathway Analysis

Twenty-four metabolites were identified as potential QRKSG biomarkers in the NS treatment at *p* < 0.05 and VIP > 1. Fifteen of the 24 metabolites were up-regulated in the QRKSG group, and nine were down-regulated than the model group (Table 1). A heat map of the 24 metabolites showed that the differentially expressed metabolites in the QRKSG and benazepril groups changed significantly (Figure 7E). Differential metabolic pathway analysis was performed using the MetaboAnalyst5.0, with an impact value > 0.1 as the potential related metabolic pathway. The identified potential biomarkers mainly involved the metabolism of amino acids in the four main pathways: D-Glutamine and D-glutamate metabolism; alanine, aspartate, and glutamate metabolism; arginine and proline metabolism; and arginine biosynthesis (Figure 7F; Table 2).

**TABLE 1:** Differentially expressed metabolites between the model and QRKSG groups.

NO.	Metabolites	RT (min)	Raw P	-log 10(p)	VIP	Trend
1	D-mannose	17.7837	5.43 × 10^–10^	9.2653	1.7753	Down
2	D-malic acid	13.3066	4.41 × 10^–9^	8.3553	1.7665	Up
3	N-acetylneuraminic acid	24.2532	1.26 × 10^–8^	7.8987	1.7606	Up
4	L-pyroglutamic acid	13.7851	1.26 × 10^–8^	7.5462	1.755	Down
5	Ornithine	17.1203	2.84 × 10^–8^	7.4335	1.753	Down
6	Pyrophosphate	15.3547	1.26 × 10^–8^	7.0296	1.745	Down
7	L-Glutamine	16.6636	3.03 × 10^–6^	5.5183	1.6969	Down
8	L-Glutamic acid	14.9244	1.53 × 10^–5^	4.8127	1.6594	Down
9	Dehydroascorbic acid	17.4486	7.77 × 10^–5^	4.1094	1.6068	Down
10	Conduritol epoxide	19.2008	1.89 × 10^–3^	2.7222	1.4287	Up
11	Nonacosane	23.4078	3.01 × 10^–3^	2.521	1.3904	Up
12	Elaidic acid	21.087	4.35 × 10^–3^	2.3615	1.3568	Up
13	D (+) altrose	20.2334	7.62 × 10^–3^	2.1182	1.2994	Up
14	Glycolic acid	7.6066	7.64 × 10^–3^	2.1168	1.2991	Down
15	Malonic acid	9.9519	8.86 × 10^–3^	2.0526	1.2825	Up
16	Succinic acid	11.0527	1.10 × 10^–2^	1.96	1.2575	Up
17	N-acetyl-D-mannosamine	19.6857	1.41 × 10^–2^	1.8495	1.2259	Up
18	Gluconic acid lactone	18.697	1.17 × 10^–2^	1.778	1.2043	Up
19	Tetracosane	22.6106	2.08 × 10^–2^	1.6818	1.1736	Up
20	1,5-anhydro-D-sorbitol	17.4806	2.24 × 10^–2^	1.6488	1.1627	Up
21	L-leucine	8.823	3.14 × 10^–2^	1.5031	1.1116	Up
22	2-deoxyribose 5-phosphate	19.9282	3.31 × 10^–2^	1.4803	1.1032	p
23	D-glucose-6-phosphate	21.8009	3.39 × 10^–2^	1.4702	1.0994	Up
24	Beta-hydroxyisovalerate	9.5753	3.91 × 10^–2^	1.4082	1.0756	Down

**TABLE 2:** Metabolic pathways of QRKSG during the treatment of NS.

NO	Pathways	Raw p	-log(p)	Impact	Label
1	D-Glutamine and D-glutamate metabolism	2.30 × 10^–3^	2.64	0.5	a
2	Alanine, aspartate and glutamate metabolism	4.90 × 10^–3^	2.31	0.31	b
3	Arginine and proline metabolism	8.43 × 10^–2^	1.07	0.2	c
4	Arginine biosynthesis	6.12 × 10^–4^	3.21	0.18	d
5	Pentose phosphate pathway	3.13 × 10^–2^	1.5	0.09	e
6	Amino sugar and nucleotide sugar metabolism	3.85 × 10^–1^	0.41	0.05	f
7	Citrate cycle (TCA cycle)	2.30 × 10^–1^	0.64	0.03	g
8	Glutathione metabolism	4.90 × 10^–3^	2.31	0.03	h
9	Nitrogen metabolism	2.30 × 10^–3^	2.64	0	i
10	Butanoate metabolism	1.50 × 10^–2^	1.82	0	j

### 3.5 Component Analysis

Component analysis using UPLC-MS/MS identified 411 compounds, including 142 flavonoids, 149 phenolic acids, 16 terpenoids, 29 alkaloids, 27 lignans, and coumarins, 3 quinones, and 45 others were identified. Thirty components were relatively regulated in the QRKSG group based on their retention time and mass spectrum (Table 3 and Supplementary Figure S1). Additionally, the size of the fan-shaped area for each compound in the fan diagram corresponded to the peak area (Supplementary Figure S2). The QRKSG group had the highest contents of Cynarin, Quercetin-4'-O-glucuronide, esculin, and Cichorium.

**TABLE 3:** UPLC-MS/MS representative information of the 30 compounds.

NO	Components	Index	RT (min)	Q1 (Da)	Class
M1	Succinic acid	mws0192	1.31	117.02	Others
M2	Esculin	mws1015	2.71	339.07	Lignans and Coumarins
M3	Chlorogenic acid	mws0178	2.74	353.09	Phenolic acids
M4	Cichoriin	Zmpn002553	2.83	339.07	Others
M5	Esculetin-7-O-glucoside	Lmbn001162	2.87	339.07	Lignans and Coumarins
M6	Cryptochlorogenic acid	mws2108	2.92	353.09	Phenolic acids
M7	11β,13-Dihydrolactucin-8-O-sulfite	Cmjp001616	3.09	359.08	Terpenoids
M8	Cynarin	mws1584	3.13	515.12	Phenolic acids
M9	Esculetin	mws1013	3.19	177.02	Lignans and Coumarins
M10	Caffeic acid	mws2212	3.38	179.03	Phenolic acids
M11	Lactucin	mws0420	3.56	277.11	Terpenoids
M12	6-Hydroxykaempferol-7-O-glucoside	pmp001309	3.65	465.1	Flavonoids
M13	Delphinidin-3-O-(6''-O-p-coumaroyl) glucoside	Lmpp003662	3.75	611.14	Flavonoids
M14	Sonchuside E	Cmjp002035	3.77	427.2	Terpenoids
M15	Quercetin-4′-O-glucuronide	Lmfn003760	3.78	477.07	Flavonoids
M16	Kaempferol-3-O-glucuronide	Lmzn001894	3.84	461.07	Flavonoids
M17	Luteolin-7-O-glucuronide	mws4167	3.91	463.09	Flavonoids
M18	Isochlorogenic acid A	pmn001382	3.92	515.12	Phenolic acids
M19	Isoquercitrin	mws0091	3.98	463.09	Flavonoids
M20	Quercetin-7-O-glucoside	mws1329	4	463.09	Flavonoids
M21	Kaempferol-7-O-glucoside	mws0089	4.15	447.09	Flavonoids
M22	8-Deoxylactucin	Cmjp002257	4.15	261.11	Terpenoids
M23	Astragalin	mws2209	4.16	449.11	Flavonoids
M24	Isochlorogenic acid B	Li512115	4.16	515.12	Phenolic acids
M25	Isorhamnetin-3-O-Glucoside	Lmjp003044	4.23	479.12	Flavonoids
M26	Baicalin	mws0052	4.33	447.09	Flavonoids
M27	Tilianin	pmp000575	5.16	447.13	Flavonoids
M28	Lactucopicrin	mws0419	5.26	409.13	Terpenoids
M29	Kaempferol	mws1068	5.68	285.04	Flavonoids
M30	Alpinetin	pmp000565	7.89	271.1	Flavonoids

### 3.6 Network Pharmacology Analysis

**FIGURE 8:**
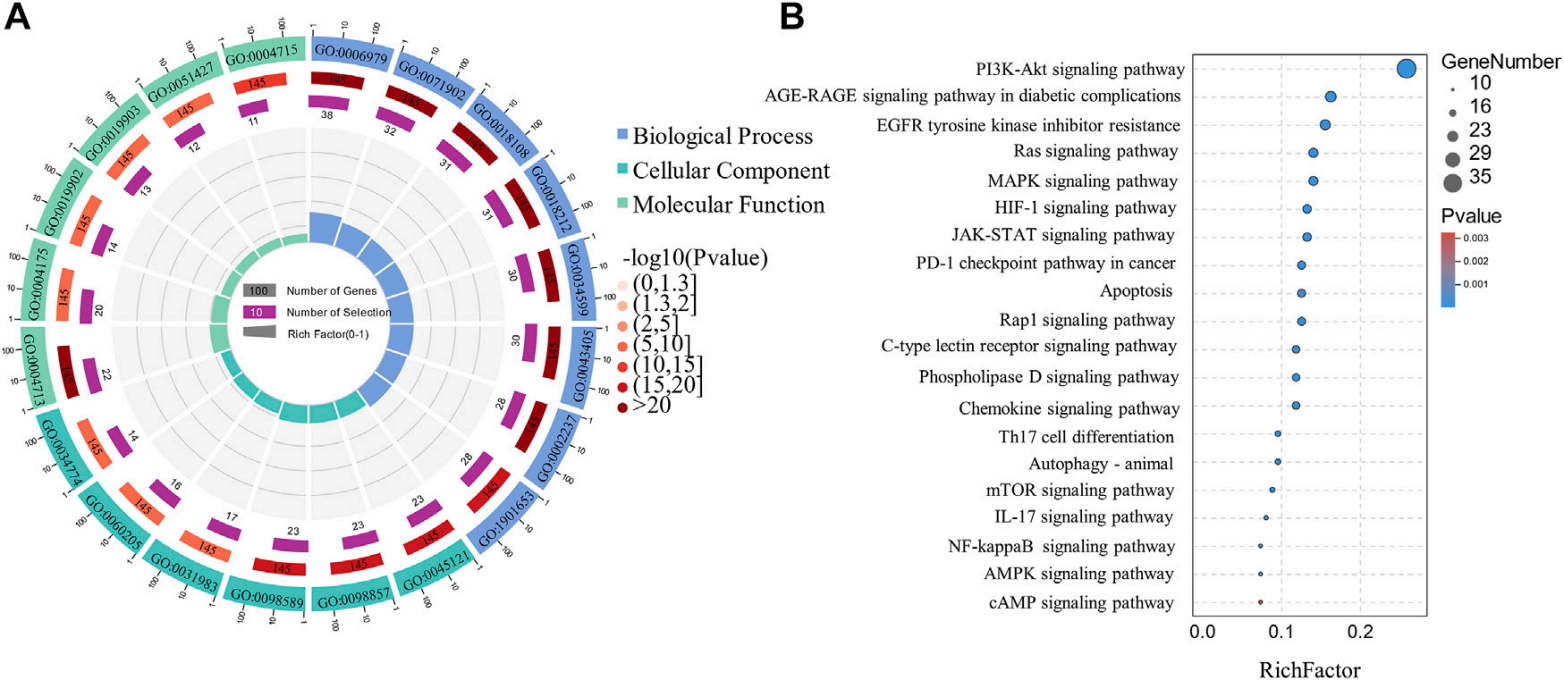
Circle and Bubble charts of GO entries and KEGG pathways. **(A)** The top 20 significantly enriched GO terms **(B)** The top 20 KEGG pathways.

The GO functional enrichment analysis yielded 2502 GO enriched entries, including 2,257 (90.2%) BPs (biological processes), 75 (3.3%) CCs (cellular components), and 170 (6.8%) MFs (molecular functions). The Omicshare tool (http://www.omicsshare.com) analyzed and visualized the GO enrichment analysis (Figure 8A) and the top 20 related, among the 153 pathways (Figure 8B). The main pathways included the PI3K-Akt signaling pathway, AGE-RAGE signaling pathway in diabetic complications, and EGFR tyrosine kinase inhibitor resistance. PI3K-Akt signaling pathway had 35 targets, including AKT1, BCL2L1, mTOR, STAT3, EGFR, and HSP90AA1. AGE-RAGE signaling pathway in diabetic complications had 22 targets, including CASP3, AKT1, TNF, MMP2, and MAPK1.

### 3.7 Metabolomics and Network Pharmacology

**FIGURE 9:**
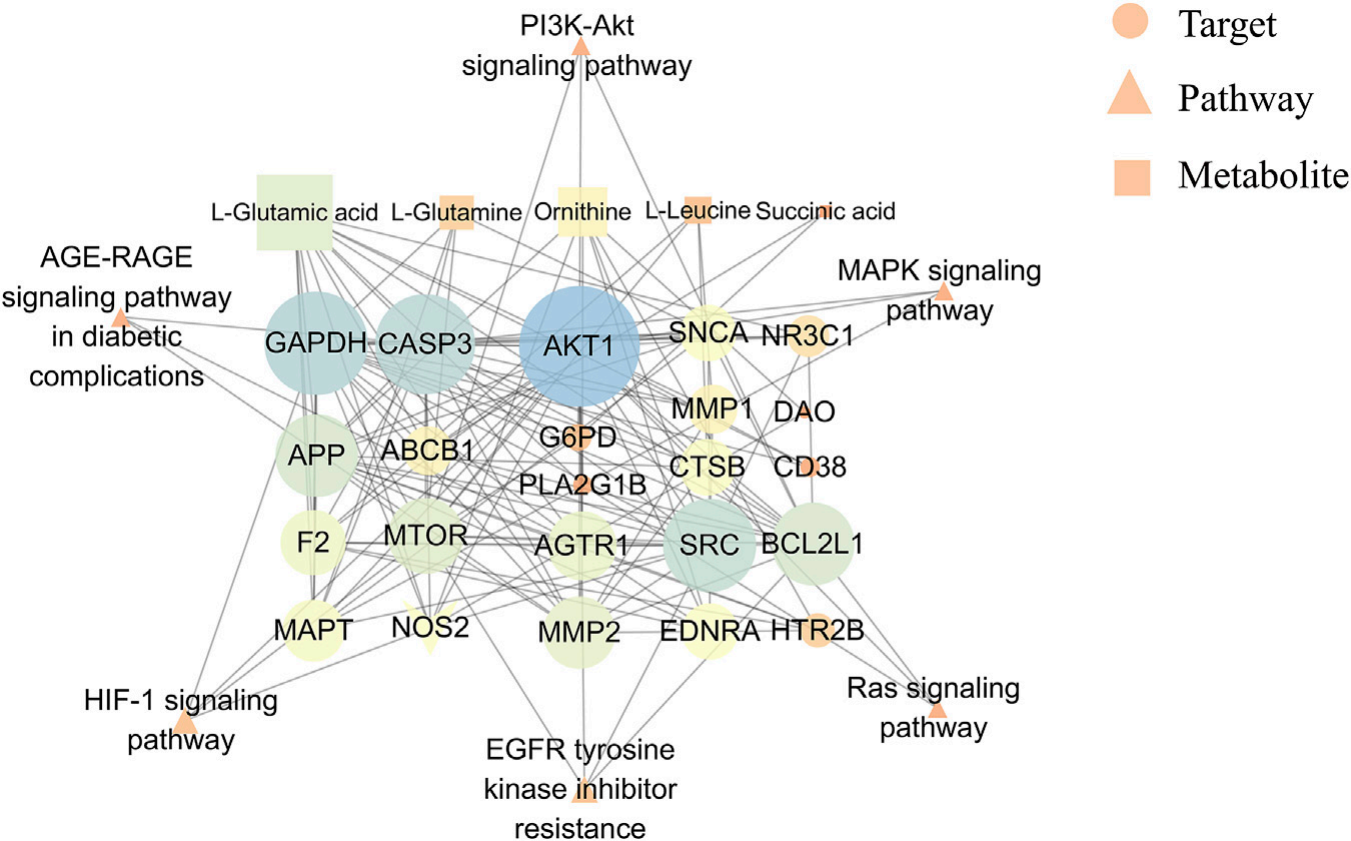
Metabolites-targets-pathways network.

The network diagram revealed 34 node interactions (6 pathways, 5 metabolites, 15 enzymes, and 8 targets) (Figure 9). The greater the degree, the more the connected edges, hence the node was more important in the network. The average degree of the candidate components was 8.52, with AKT1 (degree = 26), GAPDH (degree = 21), CASP3 (degree = 21), SRC (degree = 18), BCL2L1 (degree = 15), and mTOR (degree = 13). AKT1, BCL2L1, and mTOR were involved in the PI3K-Akt signaling pathway, protecting against ADR-induced podocyte liver damage (Dai et al., 2010). Other targets, including the apoptosis-related proteins such as CASP3 and BCL2L1 (Pradeep and Srinivasan, 2018), were related to renal tubular cell apoptosis (Ahmed et al., 2019). Therefore, AKT1, BCL2L1, CASP3, and mTOR were the core QRKSG targets for treating NS. Moreover, L-Glutamic acid and ornithine (both degree = 13) regulated multiple targets in the network diagram, indicating that QRKSG exerts therapeutic effects by regulating multiple metabolic pathways through multiple targets and metabolites.

### 3.8 Molecular Docking

**FIGURE 10:**
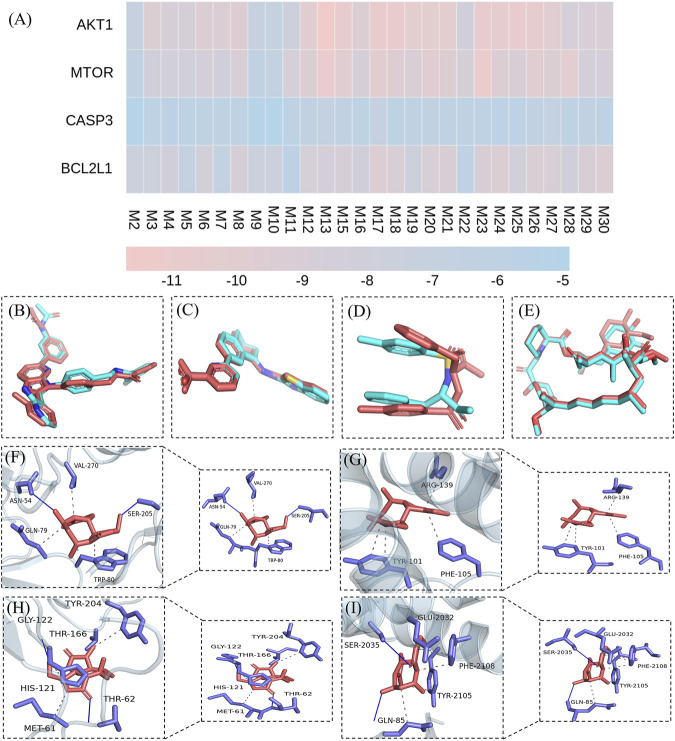
Molecular docking results. **(A)** Binding energies between active components and targets **(B)** validation of the AKTI docking solution **(C)** validation of the BCL2L1 docking solution **(D)** validation of the CASP3 docking solution **(E)** validation of the mTOR docking solution **(F)** Stable complex formed by Lactucin and AKT1 **(G)** Stable complex formed by Lactucin and BCL2L1 **(H)** Stable complex formed by Lactucin and CASP3 **(I)** Stable complex formed by Lactucin and mTOR.

First, the docking method was validated. The crystals of AKT1, CASP3, BCL2L1, and mTOR contained original ligands, including OR4 (AKT1), TQ8 (CASP3), H0Y (BCL2L1), and RAP (mTOR), respectively. The docking result **(** Figures 10B–E, blue ligand) was near-identical to the actual results (Figures 10B–E, red ligand). The root-mean-square deviation (RMSD) of the conformation of the docked and the original ligands were 0.7740, 1.6423, 0.3114, and 0.5901, respectively. These RMSD were less than 2.0 Å, indicating a feasible, effective docking scheme. In this study, molecular docking verified that the QRKSG active compounds significantly regulate AKT1, CASP3, BCL2L1, and mTOR. The smaller the absolute value of the docking score between the compound and the target, the worse their binding activity (Gupta et al., 2018). These findings revealed that AKT1, CASP3, and mTOR targets have a strong affinity for most compounds, including flavonoids, terpenoids, and phenolic acids. The absolute binding energy values between the compounds and the targets are over 4.25 (Figure 10A). The force analysis established that terpenoid Lactucin binds to AKT1 via five amino acids: ASN59, VAL-270, GLN-79, TRP-80, and SER-205 (Figure 10F), and to BCL2L1 via three amino acids: ARG-139, PHE-105, and TYR-101 (Figure 10G). To CASP3, terpenoid Lactucin binds to AKT1 via six amino acids: TYR-204, GLY-122, THR-166, HIS-121, MET-61, and THR-62 (Figure 10H), while to mTOR, binding occurs via five amino acids: GLU-2032, SER-2035, PHE-2108, TYR-2105, GLN-85 (Figure 10I).

### 3.9 Western Blotting

**FIGURE 11:**
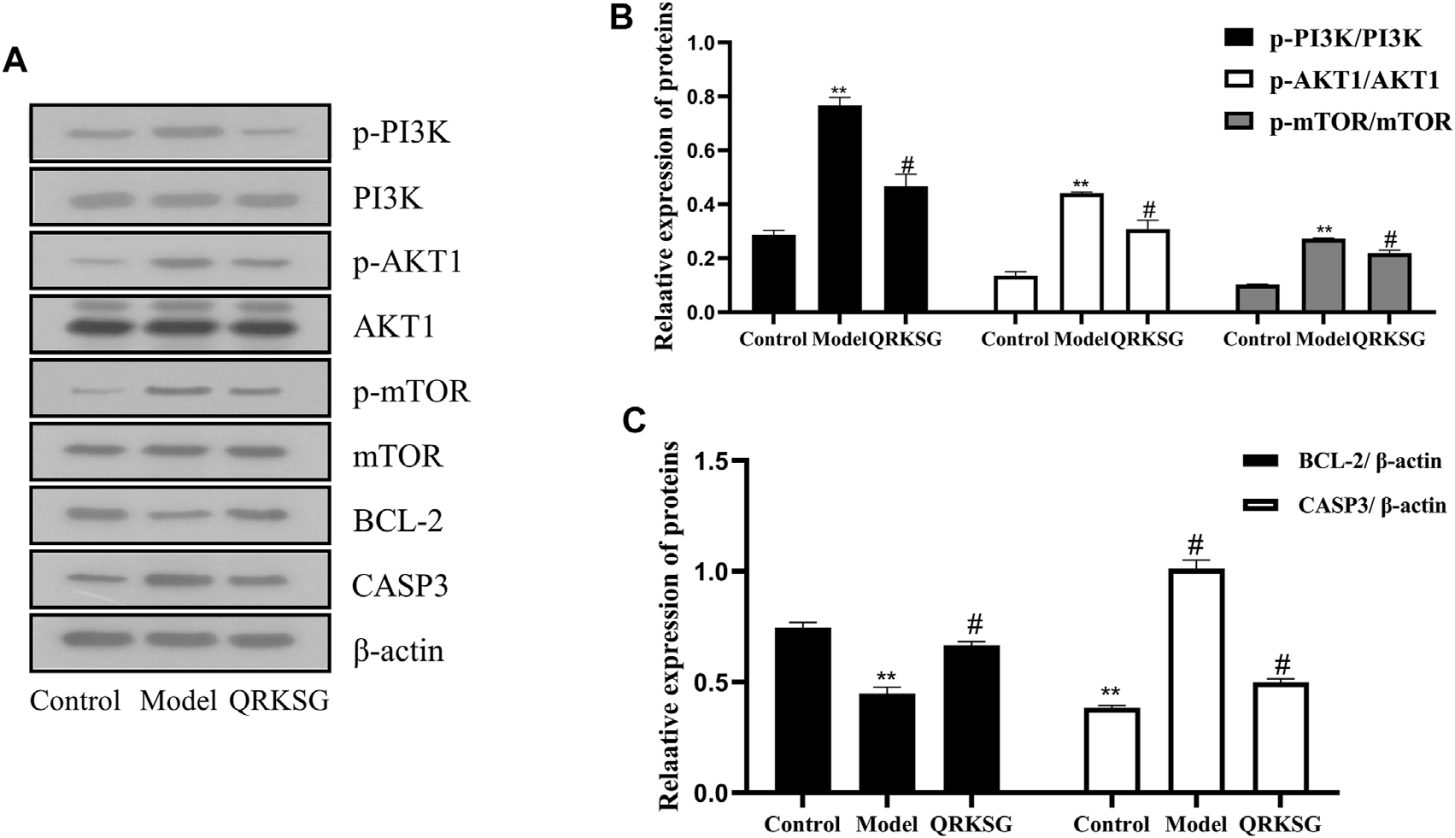
Western blot analysis of PI3K, AKT1, mTOR, BCL-2, and CASP3. **(A)** Representative images of PI3K, AKT1, mTOR, BCL-2, and CASP3 **(B)** PI3K, AKT1, and mTOR protein expressions **(C)** BCL-2 and CASP3 protein expressions. The data are presented as mean ± SD, **p < 0.05 shows comparison against the control group, while ^#^ *p* < 0.05 shows comparison against the model group.

The p-PI3K, PI3K, p-AKT1, AKT1, p-mTOR, mTOR, BCL-2, and CASP3 protein expressions were analyzed to reveal the potential mechanism of QRKSG in mediating autophagy and inhibiting apoptosis in rats with NS. The findings revealed that p-PI3K, p-AKT1, p-mTOR, and CASP3 increased, while and BCL-2 decreased in the model than the control group (Figure 11A). The greyscale quantification of each blot was performed, and the calculations of p-PI3K/PI3K, p-AKT1/AKT1, P-mTOR/mTOR, CASP3/β-actin, and BCL-2/β-actin were presented (Figures 11B,C). Besides, the expression of p-PI3K, p-AKT1, p-mTOR, and CASP3 decreased, while BCL2L1 increased in the QRKSG than the model group (*p* < 0.05). In contrast, the expression of p-PI3K, p-AKT1, p-mTOR, and CASP3 significantly increased, while BCL-2 decreased in the model than the control group (*p* < 0.05).

## 4 Discussion

Adriamycin-induced nephropathy (AN) has been extensively studied to unravel the underlying processes leading to kidney damage (Burke et al., 1977; Lu et al., 2017). In this study, the NS rat model was established by injecting ADR through the tail vein to induce NS, leading to an increased proteinuria content in the model than the control group. Renal histopathology results revealed significant morphological changes in the NS rat model manifesting as renal tubular abnormalities, dilation of kidney tubular lumen, edema, and diffuse fusion of foot processes, suggesting a successful NS modeling. The presence of proteins in the urine is a sign of podocyte damage, an indicator of glomerular filtration dysfunction, and damage of renal tubular reabsorption (Reiser et al., 2002). However, QRKSG treatment significantly reduced proteinuria, which manifested as reduced edema, morphological changes, and foot processes fusion in the renal tubes of rats in the QRKSG than the model group. Therefore, QRKSG protected rats against NS. The GC-MS metabolomics established the changes in renal metabolism, thus revealing the mechanism of QRKSG for treating NS. Twenty-four metabolic biomarkers were identified between the model and QRKSG groups. These metabolites were mainly involved in D-glutamine and D-glutamate metabolism; alanine, aspartate, glutamate metabolism; arginine and proline metabolism. The levels of L-glutamine and L-glutamate decreased after QRKSG treatment, indicating that the metabolism of D-glutamine and D-glutamate were interrupted. This observation is consistent with other findings where glutamine and phenylalanine levels were down-regulated in idiopathic NS (Sedic et al., 2014). Glutamine and glutamate are irreplaceable in D-glutamine and D-glutamate metabolism. Glutamine is catalyzed to reversibly generate glutamate, which further generates α-ketoglutarate, participating in the tricarboxylic acid (TCA) cycle to produce adenosine triphosphate (ATP) (Bharadwaj et al., 2020). Thus, contributing to material and energy production (Hakimi et al., 2016). However, glutamine plays a different role as an essential amino acid in the cell cycle progression, activating the mammalian target of rapamycin (mTOR) and homeostasis activation of reactive oxygen species (ROS) (Still and Yuneva, 2017; Bernfeld and Foster, 2019). Glutamine also regulates the mTOR pathway and coordinates various environmental factors regulating cell growth and homeostasis (Still and Yuneva, 2017; Pradeep and Srinivasan, 2018). In addition, glutamine synthetase is important in renal ammonia metabolism, maintaining the acid-base balance in the whole body, essential for optimal health (Conjard et al., 2003; Verlander et al., 2013; Harris and Weiner, 2021). In mice, glutamate inhibits the fusion of podocytes with ADR, nephropathy, cell apoptosis, renin expression and reduces urine albumin (Gu et al., 2012). Besides, glutamate converts toxic ammonia into non-toxic glutamine, preventing ammonia poisoning (Tardito et al., 2015). Therefore, QRKSG probably metabolized glutamine and glutamate, reducing the urine protein in rats with NS, thereby protecting the kidneys of these rats.

Network pharmacology analysis using UPLC-MS/MS predicted 146 targets and 153 pathways, with the PI3K-Akt signaling pathway, and the AGE-RAGE signaling pathway in diabetic complications, as the main pathways. AKT1, CASP3, BCL2L1, and mTOR were identified as potential QRKSG therapeutic targets for NS treatment. The PI3K/AKT signaling pathway is key in mammalian cell differentiation, proliferation, apoptosis, survival, autophagy and is a pivotal regulator of autophagy (Yan et al., 2018; Kim et al., 2020). PI3K phosphorylates AKT after activation, activates downstream signaling molecules such as mTOR, and exerts corresponding biological effects. mTOR is the core protein that determines the formation and maturation of autophagosomes (Li et al., 2019). mTOR activation in the kidney promotes up-regulation of pro-inflammatory and pro-fibrotic factors, leading to tubular interstitial fibrosis and atrophy (Lieberthal and Levine, 2012). In contrast, inhibiting mTOR activation protects the kidneys against podocyte apoptosis (Lee et al., 2015). Inhibiting the PI3K/Akt/mTOR pathway induces autophagy (Tsai et al., 2015), promoting cell health in response to various cell stresses and nutritional conditions and subsequently protecting cells from damage (Kitada et al., 2017). For example, mTOR activation negatively regulates autophagy in several types of cells (Lindqvist et al., 2015; Liu et al., 2018). Autophagy is key in preventing kidney diseases because it maintains homeostasis and the integrity of renal tubules (Peng et al., 2019). Thus, up-regulating autophagy is an efficient treatment strategy for kidney diseases (Bhatia and Choi, 2020). Besides, autophagy substantially reduced renal inflammation and interstitial damage in a unilateral ureteral obstruction (UUO) model where mTOR inhibition significantly increased autophagy and suppressed UUO-induced inflammation (Du et al., 2017). In the model group, PI3K, AKT1, and mTOR expression levels increased in the QRKSG than the control group, indicating that the PI3K/Akt/mTOR signaling pathway was activated. However, QRKSG treatment inhibited the PI3K/Akt/mTOR signaling pathway, which indicates that QRKSG probably increased autophagy to prevent renal tubular interstitial fibrosis and atrophy.

Bax, a member of the BCL-2 family, is up-regulated by p53 transactivation, while p53 transcriptionally suppresses BCL-2, thereby activating caspase-9 and subsequently caspase-3 (Wen et al., 2014; Wnek et al., 2017). Increasing the ratio of BCL-2 to Bax achieves the anti-apoptotic function of BCL-2 (Castro-Gonzalez et al., 2021), while the activation of caspase-3 promotes cell apoptosis (Sun et al., 2011). In addition, Srinivasan and Pradeep established that increasing the apoptosis mediator Bax decreased BCL-2 in the kidneys of diabetic rats (Pradeep and Srinivasan, 2018). Parallel to the present study, Osama et al. reported that paricalcitol and enalapril protective cells against streptozotocin (STZ)-induced renal tubular cell apoptosis by reducing the caspase-3 expression and increasing BCL-2 expression (Pradeep and Srinivasan, 2018). Wu et al. also established that Fang Qi Dan down-regulates the expression of Bax protein while increasing the BCL-2 protein, which protects podocytes, and restores the selective filtration function of the glomerulus (Wu et al., 2014). The expression of CASP3 increased in the model group, while the expression level of BCL2L1 decreased (VS control group), indicating the increased ability of renal cell apoptosis. However, QRKSG treatment weakened the renal apoptotic capacity. These results indicate that QRKSG protects renal tubular cells against ADR-induced renal damage. The findings are consistent with previous research (Lieberthal and Levine, 2009; Kaushal et al., 2020; Zhou et al., 2020; Bhosale et al., 2021; El-Gowily et al., 2021), and the relevant target graphs are shown in Supplementary Figure S3. Therefore, QRKSG could promote autophagy and anti-apoptosis through the expression of AKT1, CASP3, BCL2L1, and mTOR, thereby conferring protection to the podocytes and maintaining renal function and tubular homeostasis.

Molecular docking revealed the mode of interaction between the active QRKSG components and target proteins in the kidneys, where most components are bound to core targets (AKT1, CASP3, BCL2L1, and mTOR). The comprehensive evaluation of the docking results initially revealed the QRKSG material basis of treating NS. This observation has profound implications for exploring the molecular-level QRKSG mechanism of action.

## 5 Conclusion

**FIGURE 12:**
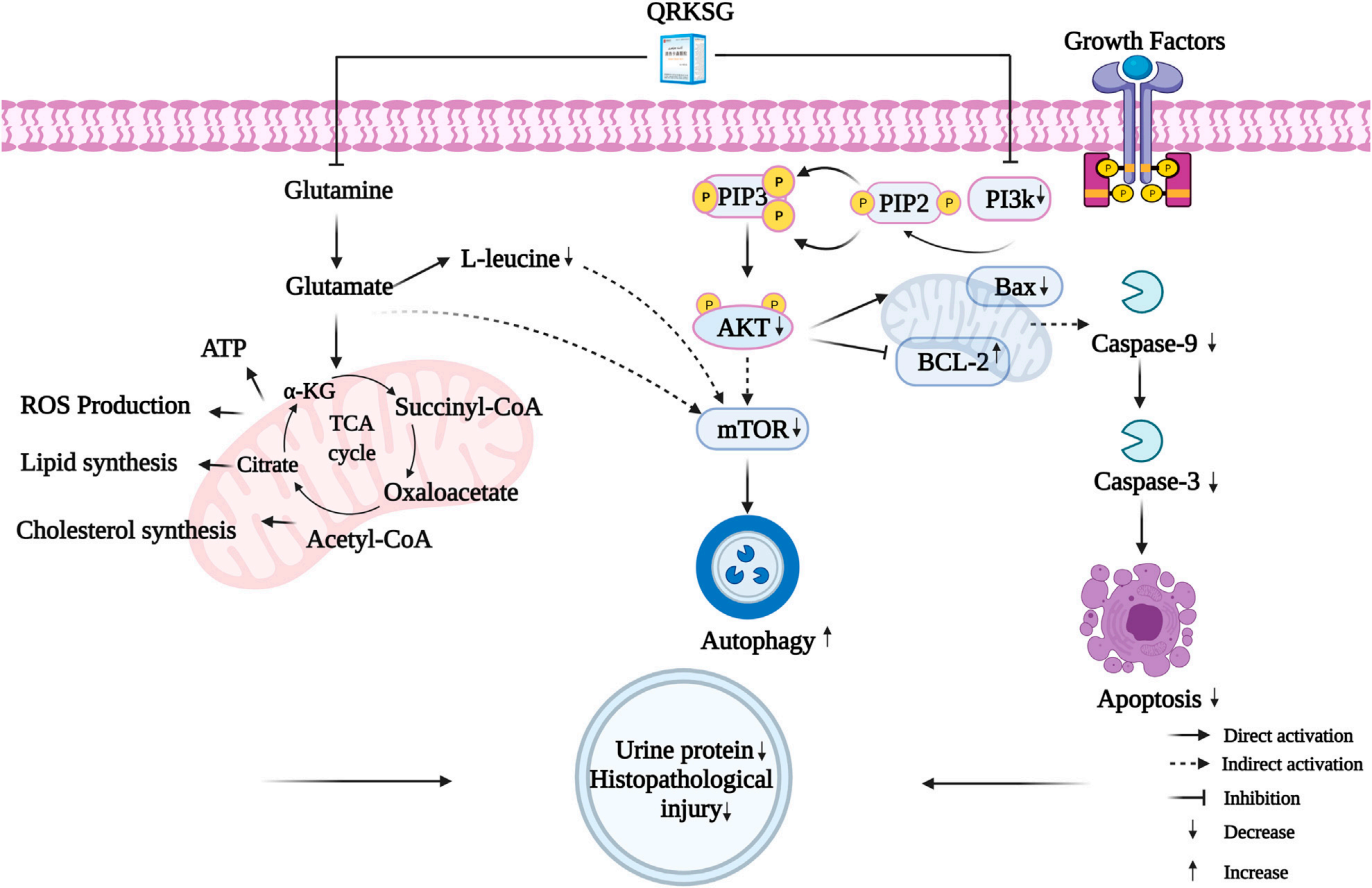
The molecular mechanisms of QRKSG in treating NS.

A comprehensive analysis revealed 23 key targets related to five metabolites and six pathways. These potential targets were related to multiple QRKSG active components. Among the key targets, AKT1, MTOR, CASP3, and BCL2L1 were important for the QRKSG underlying mechanism in treating NS. Specifically, the potential QRKSG mechanisms include inhibiting cell apoptosis and increasing autophagy, D-Glutamine, and D-glutamate metabolism, reducing proteinuria and weakening kidney damage (Figure 12). Therefore, these findings on the potential underlying QRKSG mechanism provide a theoretical basis for QRKSG clinical application.

## Data Availability

The original contributions presented in the study are included in the article/Supplementary Material, further inquiries can be directed to the corresponding authors.

## Supplementary Material

Supplementary Figure S1 Extraction ion chromatogram of each QRKSG component.

Supplementary Figure S2 The peak area distribution from the 30 compounds identified in QRKSG

Supplementary Figure S3 Graphical abstract.

## Glossary

ADR: Adriamycin
AKT1: AKT Serine/Threonine Kinase 1
ALB: albumin
AN: Adriamycin-induced nephropathy
ATP: adenosine triphosphate
BAX: BCL2 Associated X, Apoptosis Regulator: BCL2 Associated X, Apoptosis Regulator
BCA: Bicinchoninic Acid
BCL2: Apoptosis Regulator
BAX: BCL2 Associated X, Apoptosis Regulator: BCL2 Associated X, Apoptosis Regulator
BPs: biological processes
BUN: urea nitrogen
CASP3: Caspase 3
CCs: cellular components
COX-2: Cyclooxygenase-2
Cr: creatinine
CUR: 25psi
ECL: enhanced chemiluminescence
EGFR: Epidermal growth factor receptor
ESI: electrospray ionization source
GAPDH: Glyceraldehyde-3-phosphate dehydrogenase
GC-MS: Gas chromatography–mass spectrometry
GO: gene ontology
GSI: 50psi
GSII: 60psi
HPLC: High-performance liquid chromatography
HRP: horseradish peroxidase
HSP90AA1: Heat shock protein HSP 90-alpha
KEGG: Genes and Genomes
MFs: molecular functions
MSTFA: N-Methyl-N-(trimethylsilyl)trifluoroacetamide
mTOR: Mechanistic Target of Rapamycin Kinase
PCA: principal component analysis
PGE2: prostaglandin E2
PI3K: Phosphoinositide-3-kinase
PLS-DA: partial least squares discriminant analysis
PVDF: polyvinylidene fluoride
ROS: reactive oxygen species
SD: Sprague Dawley
SRC: Proto-oncogene tyrosine-protein kinase Src
STAT3: Signal transducer and activator of transcription 3
STZ: streptozotocin
TBST: Tris-buffered saline, 0.1% Tween 20
TCA: tricarboxylic acid
TCM: traditional Chinese medicines
TG: total triglycerides
TIC: total ion current chromatograms
TMCS: Trimethylchlorosilane
UPLC-MS/MS: Ultra performance liquid chromatography-tandem mass spectrometer
VIP: variable importance in the projection
UUO: unilateral ureteral obstruction
3D: three-dimensional
