# Supplementary figures and images for: Integrated Metabolomics and Network Pharmacology to Establish the Action Mechanism of Qingrekasen Granule for Treating Nephrotic Syndrome
Source: Front Pharmacol. 2021 Dec 6;12:765563. doi: 10.3389/fphar.2021.765563 (PMC8685401; doi:10.3389/fphar.2021.765563)

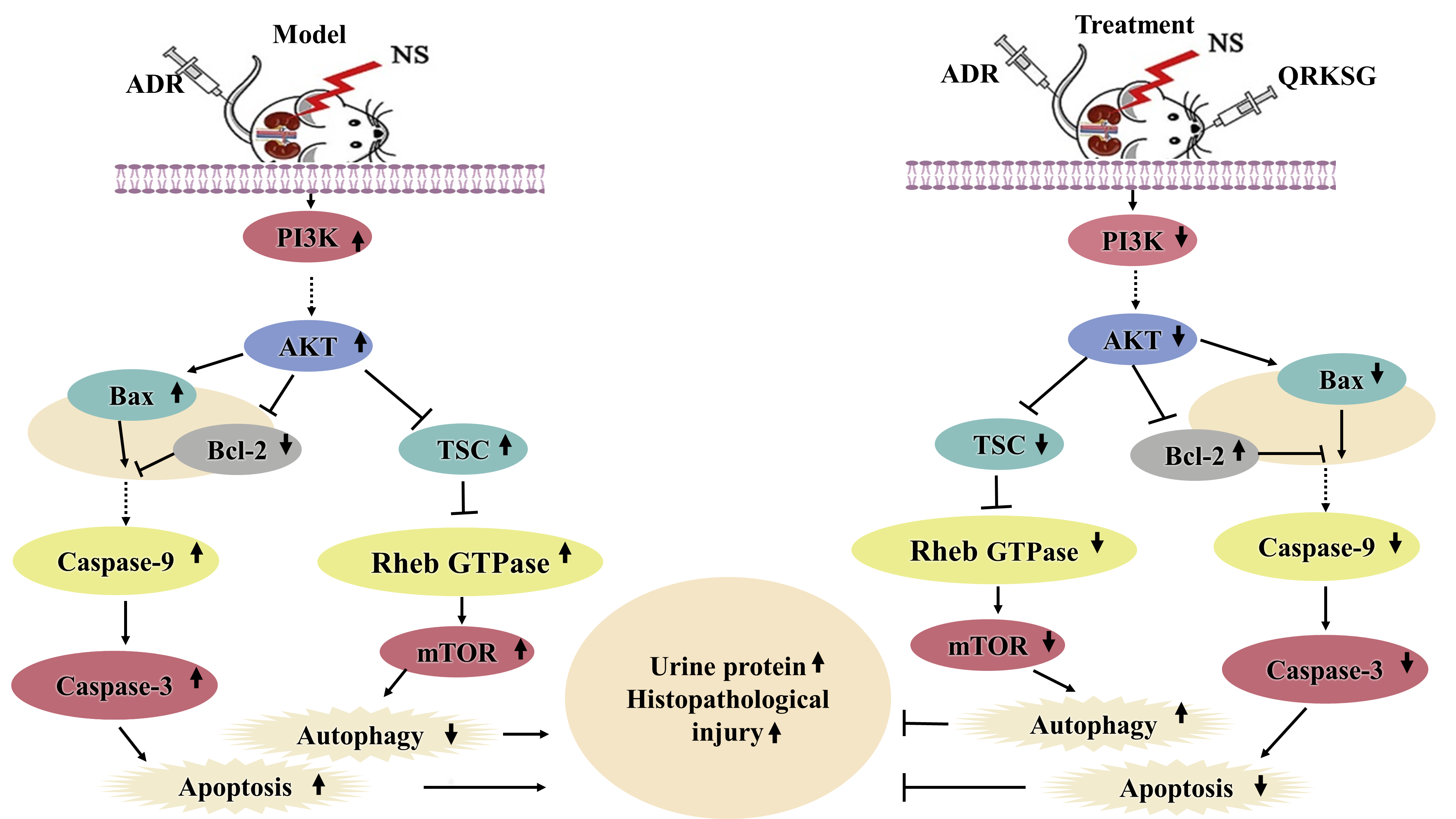

Supplement: Supplementary file 1 [file Image3.TIF]

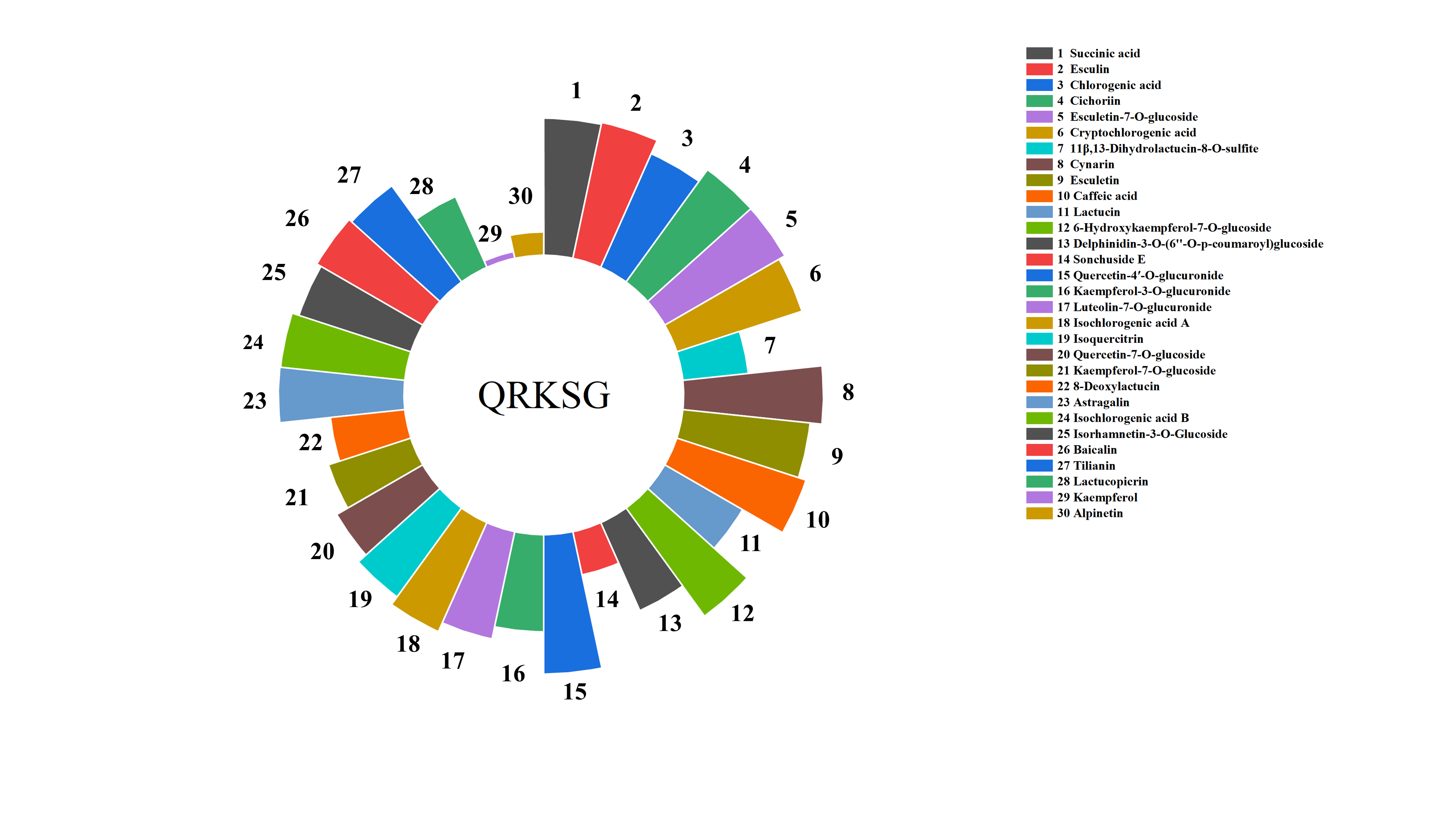

Supplement: Supplementary file 2 [file Image2.TIF]

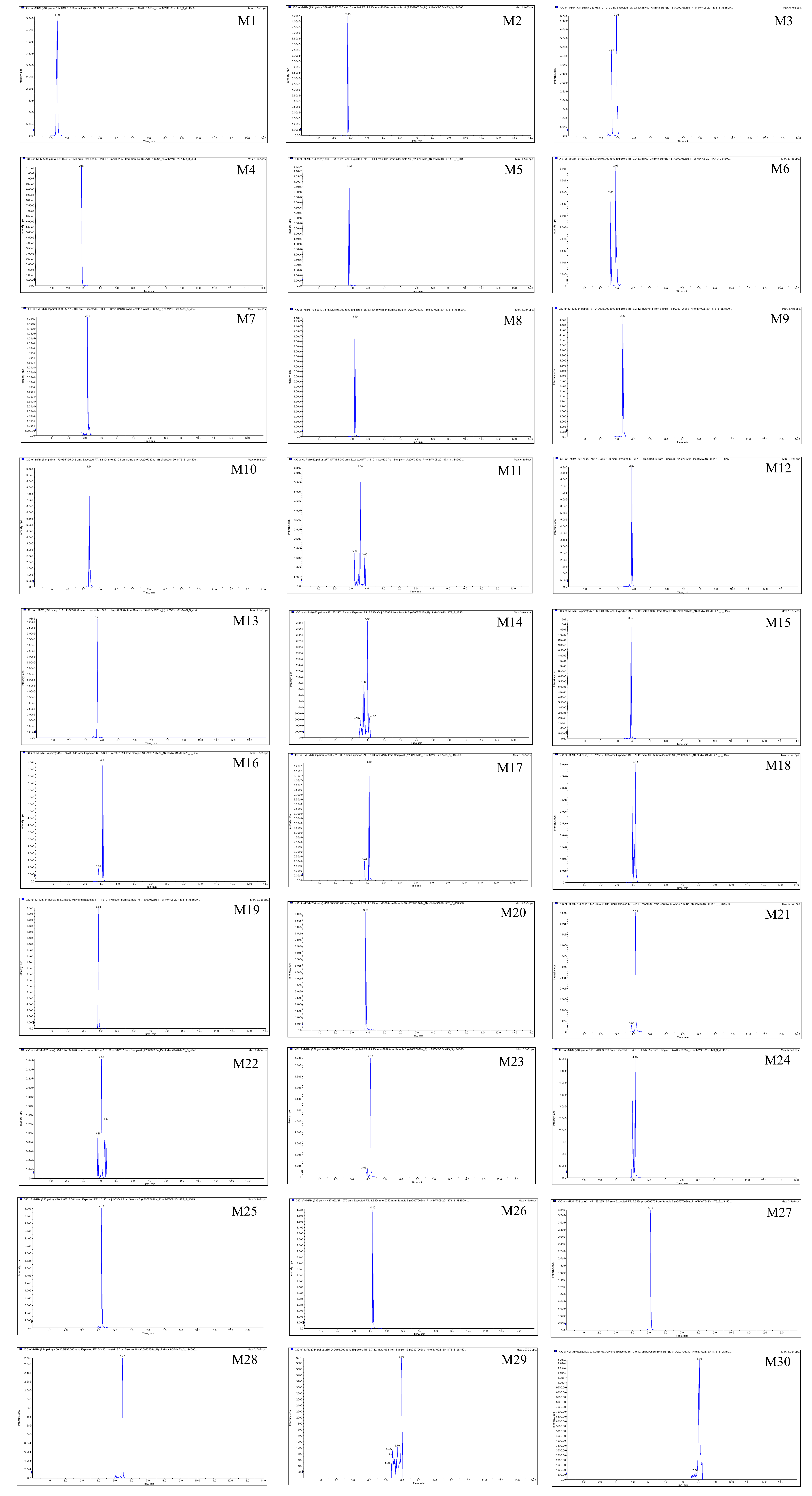

Supplement: Supplementary file 3 [file Image1.TIF]
