# Supplementary material for: Integrated Metabolomics and Network Pharmacology to Establish the Action Mechanism of Qingrekasen Granule for Treating Nephrotic Syndrome
Source: Front Pharmacol. 2021 Dec 6;12:765563. doi: 10.3389/fphar.2021.765563 (PMC8685401; doi:10.3389/fphar.2021.765563)
Supplement: Supplementary file 4 [file DataSheet1.docx]

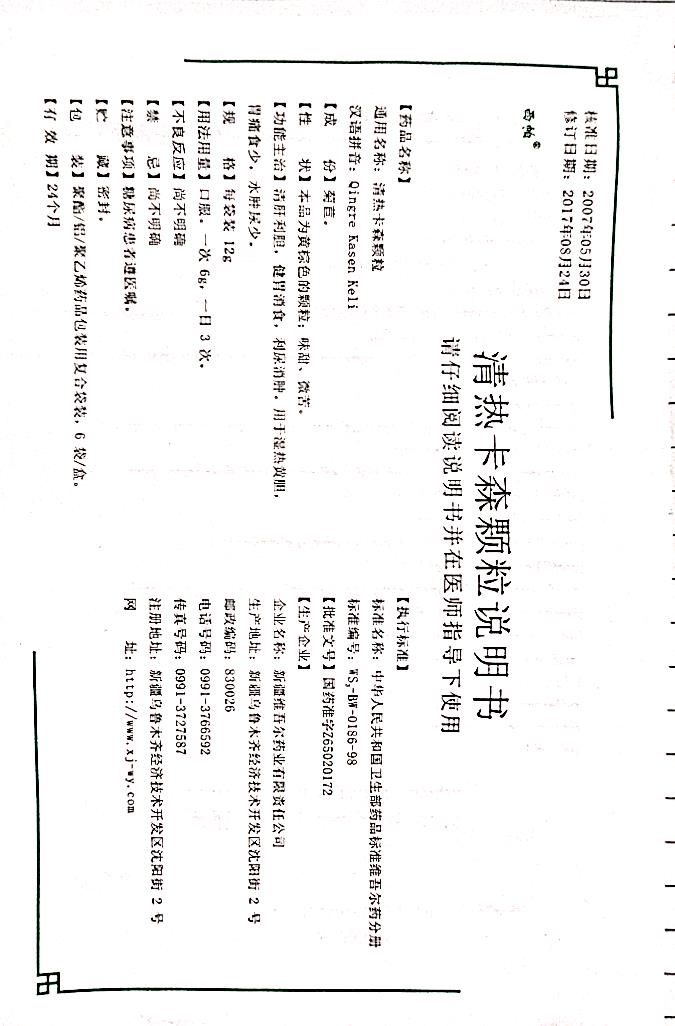
Annex 1. Detailed information and clinical instructions for the use of QRKSG.


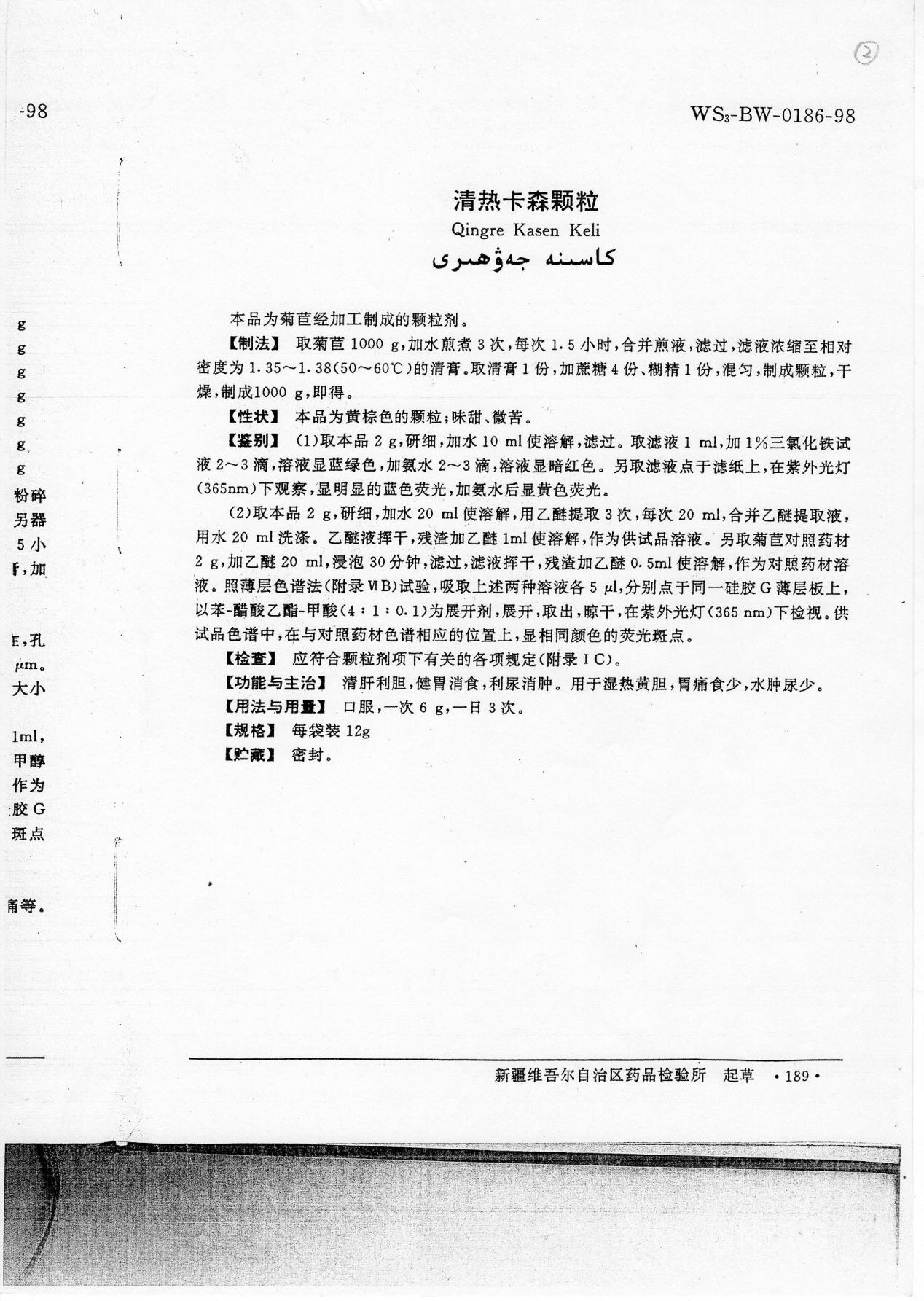
(Only used as a reply to the editor, not in the scope of the manuscript)

Annex 2. Representative micrographs of histopathology in each group at magnification ×200. Black arrows represent kidney tubules, and red arrows represent glomeruli. (A) control group (B) model group (C) benazepril group (D) QRKSG medium-dose group (E) QRKSG low-dose group (F) QRKSG high-dose group.


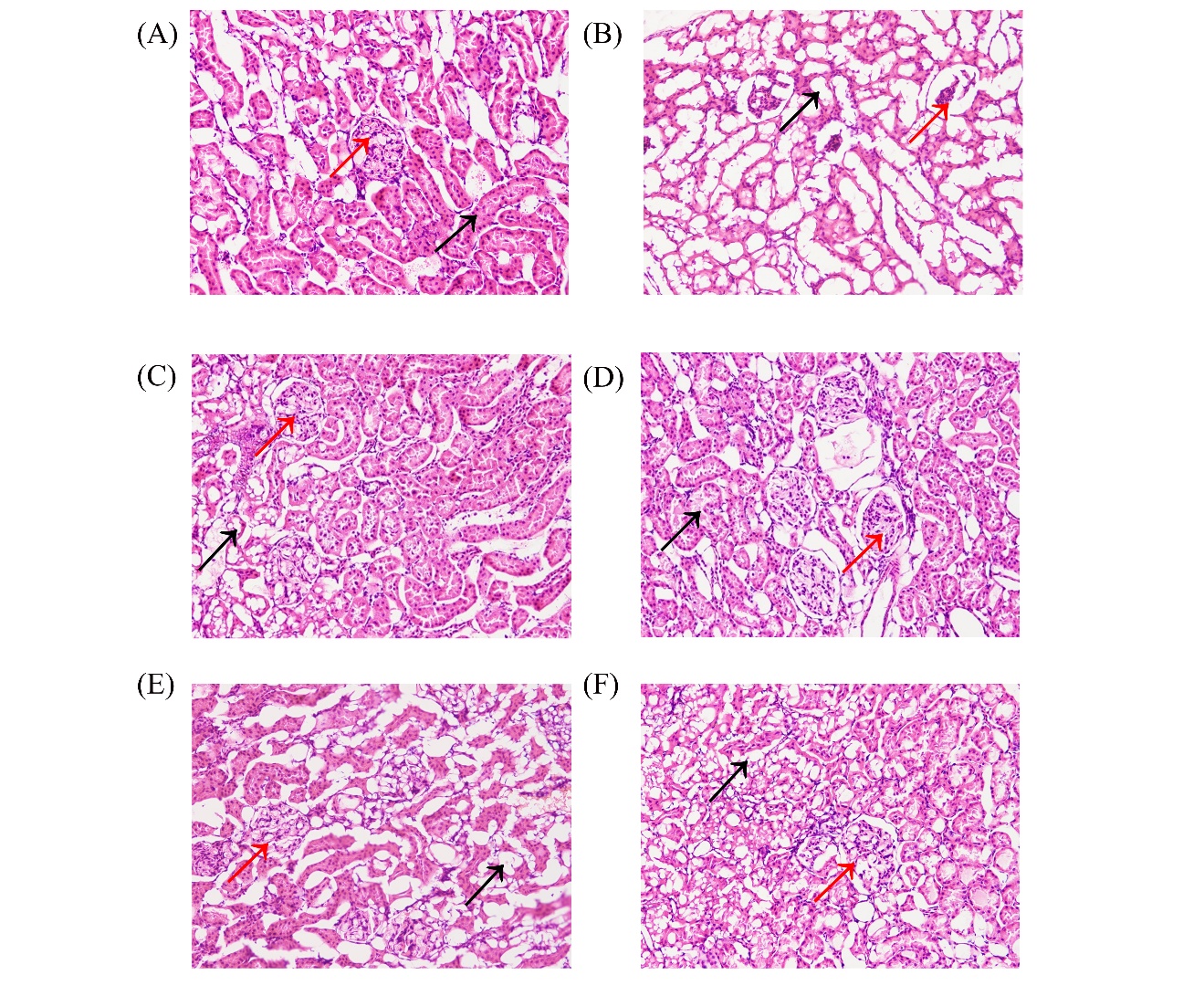
(Only used as a reply to the editor, not in the scope of the manuscript)
